# Relationship between valued life activity and brachial–ankle pulse wave velocity from the Wakayama study: A cross-sectional study

**DOI:** 10.1097/MD.0000000000046764

**Published:** 2025-12-19

**Authors:** Katsushi Yokoi, Nobuyuki Miyai, Masato Sakaguchi, Toshifumi Oka, Miyoko Utsumi, Megumi Nakamura, Mikio Arita

**Affiliations:** a Graduate School of Rehabilitation Science, Osaka Metropolitan University, Habikino City, Osaka, Japan; b Wakayama Medical University School of Health and Nursing Science, Wakayama City, Wakayama, Japan; c Sumiya Rehabilitation Hospital, Wakayama City, Wakayama, Japan; d Wakayama Faculty Nursing, Tokyo Healthcare University, Wakayama City, Wakayama, Japan.

**Keywords:** a cross-sectional population-based study, arterial stiffness, brachial–ankle pulse wave velocity, community-dwelling people, valued life activity

## Abstract

Arterial stiffness is strongly associated with atherosclerosis and is a major cause of cardiovascular events, making prevention essential for middle-aged adults. Since lifestyle habits considerably influence arterial stiffness, it is crucial for middle-aged and older adults to understand how to maintain their health through daily activities. This study investigated the relationship between the characteristics of valued life activities (VLAs) and brachial–ankle pulse wave velocity (baPWV), which reflects arterial stiffness. A cross-sectional analysis was conducted on 380 community-dwelling individuals surveyed in 2017 as participants in the Wakayama study. Participants completed a self-administered questionnaire on VLAs, and their baPWV was measured. The questionnaire assessed the number, domain, frequency, continuity, performance, and satisfaction of VLAs. Among the participants, 293 (77.1%) had a baPWV ≥ 14 m/s, indicating a cardiovascular risk level. In the main analyses, having 3 VLAs were significantly associated with lower odds of baPWV ≥ 14 m/s (adjusted odds ratio [95% confidence interval]: 0.33 [0.13–0.85]). The crude odds ratio for productivity-only activities was 2.54 (95% confidence interval: 0.87–7.41), while participation in both leisure and productivity activities was associated with lower odds (0.64 [0.39–1.04]), particularly among women (0.49 [0.26–0.91]). In sensitivity analyses that included age adjustment, these associations were attenuated and no longer statistically significant, reflecting the strong influence of age on arterial stiffness. Nevertheless, the findings suggest that the number and type of VLAs, especially productivity and leisure activities among women, may be relevant factors concerning arterial stiffness. Assessing VLAs could therefore be beneficial in designing health support programs aimed at addressing arterial stiffness for middle-aged and older adults.

## 1. Introduction

Cardiovascular disease (CVD) is the leading cause of death worldwide.^[1]^ Atherosclerosis is a key predictor of CVD, and arterial stiffness is strongly associated with it.^[2]^ Brachial–ankle pulse wave velocity (baPWV) is an independent prognostic indicator of CVD; a 1 m/s increase in baPWV raises the incidence of CVD by 12%.^[3]^ PWV is a noninvasive method for assessing arterial stiffness and is an indicator of vascular damage; it reflects arterial stiffness and blood pressure, which are major risk factors for CVD.^[4]^ The baPWV is commonly used as an indicator of arterial stiffness in clinical practice, and the propagation velocity tends to increase with arterial stiffness.^[5]^ Improving arterial stiffness is important for the primary prevention of cardiovascular events. Physical inactivity is a risk factor for cardiovascular events,^[6]^ and daily physical activity and leisure time are important for preventing CVD.^[7]^ Eliminating physical inactivity is estimated to reduce the prevalence of major non-communicable diseases, including CVD, type 2 diabetes, and breast and colon cancers, by 6% to 10%, and to increase overall life expectancy.^[6]^ Moreover, even modest engagement in leisure-time physical activity, such as 10 to 36 minutes per day, is associated with a substantially lower risk of CVD, with an age-adjusted risk ratio of 0.71 compared to only 0 to 9 minutes per day.^[7]^ Light to moderately intense physical activity is associated with reduced arterial stiffness, and less physical activity may worsen it.^[8]^

Arteriosclerosis is greatly influenced by lifestyle factors, including daily activities and diet. Although this has been discussed in terms of physical activity and exercise, it has not yet been examined from the perspective of daily activities. Various terms have been used to describe the activities individuals consider important for health and disease. For example, meaningful activities are defined as “physical, social, and leisure activities tailored to the person’s needs and preferences.”^[9]^ Conversely, valued life activities (VLAs) are those activities an individual considers particularly important or meaningful and are focused on high-priority and important activities.^[10]^ In occupational therapy, which deals with the health of local residents and people with disabilities, the word “occupation” is used to express meaning and purpose in life.^[11]^ Meaningful activities or occupations lack a clear definition, resulting in a broad interpretation of meaningfulness.^[12]^ In this study, we focused on activities that are considered particularly important in life, referring to them as “VLAs.” A VLA is any activity that an individual finds particularly pleasurable or meaningful, including self-care tasks, social interactions, and leisure interests.^[10]^

The inability to participate in VLAs has been associated with negative perceptions of health status, reduced quality of life, and psychological distress.^[13,14]^ VLAs are based on needs and preferences and personal endorsement of the core values of their activities, making them a part of their lifestyle.

Since VLAs contribute to people’s health and well-being, it is important to consider people not only from the perspective of physical activity but also from VLAs. In particular, as atherosclerosis progresses asymptomatically from middle age, early prevention is required.^[15]^ Research on VLAs and health has focused on specific diseases rather than community populations.

This cross-sectional study aimed to investigate the relationship between VLA characteristics and PWV among community dwellers. Viewing arteriosclerosis through the lens of VLAs may enhance the perception of it as a personal concern.

## 2. Methods

### 2.1. Study design

This cross-sectional study was part of the health examinations for arteriosclerosis in Japan.

This cross-sectional study was designed and reported in accordance with the Strengthening the Reporting of Observational Studies in Epidemiology guidelines.

### 2.2. Study participants

The study population comprised individuals who underwent arteriosclerosis health examinations in Katsuragi, Wakayama Prefecture. Eligibility criteria required that participants not be hospitalized or institutionalized, be able to ambulate independently, and be able to visit the health checkup site. The inclusion criterion was the absence of a dementia diagnosis, while the exclusion criterion was difficulty in completing the questionnaire due to hearing or visual impairments. Of the 665 respondents to the VLAs questionnaire, we used the data of 381 with available baPWV values from the Wakayama study conducted in September 2017. We analyzed data from 380 participants and excluded those whose PVW was not measured (n = 1).

This study was approved by the Wakayama Medical University Ethics Committee (approval number: 92). Written informed consent was obtained from all participants. All procedures were conducted in accordance with the principles of the Declaration of Helsinki.

### 2.3. Characteristics of VLAs

The self-administered questionnaire on the characteristics of VLAs comprised the following items: name of VLAs; name of the most important activity; and domain (self-care, productivity, or leisure). To identify the most important activity, participants answered questions on performance (ranging from “very good performance” [10 points] to “poor performance” [1 point]); satisfaction (ranging from “very good satisfaction” [10 points] to “low satisfaction” [1 point]); frequency (daily, weekly, monthly, yearly); and continuity (<1, 1–5, and > 5 years). VLA is defined as “an activity an individual deems particularly important or meaningful,”^[10]^ emphasizing individual subjectivity. This concept is grounded in the notion of idiosyncratic functional goals, specifically, personally unique functional objectives that shape the activities valued and how health states are evaluated.^[16]^ While this inherently introduces heterogeneity, it also reflects the essence of VLAs. To ensure measurement validity, the present questionnaire was developed with reference to the Canadian Occupational Performance Measure (COPM), a tool with established reliability and validity across diverse populations. Participants were limited to a maximum of 3 VLAs, based on previous studies.^[10]^ The VLAs, performance, and satisfaction domains were based on the COPM.^[17]^ According to the participants’ responses, the VLA domains were categorized into self-care, productivity, and leisure. The COPM has been widely used across populations, including older adults, and has demonstrated adequate reliability and validity.^[17]^ Since the present questionnaire was developed based on the COPM framework and aimed to identify individually meaningful VLAs rather than function as a psychometric scale, no separate reliability or validity testing was performed for this study.

The questionnaire was administered face-to-face by an occupational therapist in a separate booth at the health examination site. Participants were also asked about their sex, age, underlying medical conditions, smoking status, and alcohol consumption.

### 2.4. baPWV

Following blood pressure measurement, the participants remained in the supine position for baPWV measurement by a physician or nurse using the BP-203RPE II or III form PWV or ankle–brachial index (Omron Colin Co. Ltd., Tokyo, Japan).^[18]^ Occlusion cuffs connected to a plethysmograph and oscillometric sensors were attached to the participants’ upper arms and ankles. The cuffs used were based on the size of the individual’s arm and ankle circumference (3 arm cuff sizes: large, 30–38 cm; medium, 20–32 cm; small, 16–25 cm; and 1 ankle cuff size, 16–33 cm). Waveforms were measured using volume plethysmograph sensors placed on the occlusion cuffs of the upper arm and ankle, using the time interval (ΔTba) between the wavefronts of the upper arm and ankle. The lengths of the paths from the suprasternal notch to the upper arm (Lb) and ankle (La) were automatically obtained based on the individual’s height. The baPWV was calculated using the formula: (La − Lb)/ΔTba. This device provides highly reproducible measurements and correlates well with aortic PWV measured using a catheterized manometer.^[19]^ Before measuring baPWV, participants’ weight and height were measured.

### 2.5. Statistical analysis

The *t* test, Mann–Whitney *U* test, or chi-square test was used to assess the differences in baPWV below and above 14 m, according to the participants’ characteristics and the characteristics of the VLAs. Shapiro–Wilk test was used to investigate normality. Participants with a baPWV ≥ 14 m/s were considered as having a high risk of arteriosclerosis.^[20]^ This threshold corresponds to a moderate risk in the Framingham risk score^[21]^ and suggests an increased risk of developing hypertension, making it a recommended cardiovascular risk level for lifestyle-related improvement. In addition, since higher thresholds of 16 to 18 m/s are more commonly applied in clinical practice,^[4]^ we performed sensitivity analyses with these alternative cutoffs to examine the robustness of our findings. We conducted univariate analyses to explore the associations between each variable and arterial stiffness. Variables that showed statistically significant associations in the univariate analyses (*P* < .05), along with those deemed clinically relevant, were included in the multivariate logistic regression models. These variables were entered into the multivariate logistic regression models using the forced entry method in an exploratory manner. The odds ratios for the association between the characteristics of VLAs and baPWV were calculated using multivariate logistic regression analysis. We established the bivariate dependent variables of baPWV > 14 m/s and < 14 m/s associated with an increased risk of cardiovascular events and the characteristics of VLAs as independent variables. The number of VLAs was categorized as < 3 or 3, the frequency as daily, weekly, monthly, or yearly, and the duration as < 1 year and > 1 year. The analysis models were Model 1, in which adjustments were made for the characteristics of VLAs; Model 2, in which adjustments were made for sex in addition to the Model 1 variables; and Model 3, in which adjustments were made for body mass index, alcohol intake, smoking status, and underlying diseases, in addition to the Model 2 variables. To avoid over-adjustment, we did not perform adjustments for age.^[22]^ Additionally, as a sensitivity analysis, we constructed a model that included age as a covariate to examine the robustness of the findings. Crude odds ratios for the domains of VLAs were calculated independently. The VLA domains were classified into 7 patterns: self-care only, productivity only, leisure only, productivity and leisure, self-care and productivity, self-care and leisure, and productivity and leisure. In this study, the left and right baPWV values were significantly correlated at 0.92 (*P* < .01), and similar to a previous study, the mean left and right baPWV values were considered representative values.^[18]^ The variance inflation factors (VIFs) for the performance score (2.45) and satisfaction score (2.46) were both below 10, indicating no multicollinearity.

## 3. Results

Of the 380 participants, 157 were men and 223 were women, with a mean age of 69.5 ± 10.2 years. Among them, 87 (22.9%) had a baPWV < 14 m/s, while 293 (77.1%) had a baPWV > 14 m/s (Table 1). VLAs are diverse, ranging from self-care, such as cooking, making lunch, bathing, and putting on makeup; leisure activities, such as playing musical instruments, singing, traveling, and walking; and productive activities, such as farm work, employment, political activities, and community activities. The characteristics of the VLAs and baPWVs are listed in Table 2.

**Table 1:** Participant characteristics.

	All	baPWV < 14 m/s	baPWV ≥ 14 m/s	*P*-value
	n = 380	n = 87	n = 293	
Age (yr)	69.4 (10.2)	60.1 (11.4)	72.2 (7.9)	<.01
Male	157 (41.3)	34 (39.1)	123 (42.0)	.63
Body mass index (kg/m^2^)	22.9 (3.3)	23.0 (3.9)	22.9 (3.1)	.86
Smoking	30 (7.9)	4 (4.6)	26 (8.9)	.19
Alcohol intake	153 (40.3)	39 (44.8)	114 (38.9)	
Daily	70 (18.4)	16 (18.4)	54 (18.4)	.43
Weekly	83 (21.8)	23 (26.4)	60 (20.5)	
Systolic blood pressure (mm Hg)	133.2 (17.5)	121.0 (14.8)	136.8 (16.6)	<.01
Diastolic blood pressure (mm Hg)	78.5 (10.3)	75.3 (10.1)	79.4 (10.2)	<.01
baPWV				
Right	1667.0 (370.9)	1256.6 (123.6)	1788.8 (330.2)	
Left	1662.8 (356.3)	1265.0 (114.8)	1780.9 (315.8)	
Mean	1664.9 (356.0)	1260.8 (112.1)	1784.8 (312.6)	
Diabetes	42 (11.1)	6 (6.9)	36 (12.3)	.13
Hypertension	176 (46.3)	20 (23.0)	156 (53.2)	<.01
Dyslipidemia	115 (30.3)	13 (14.9)	102 (34.8)	<.01
Heart disease	44 (11.6)	5 (5.7)	39 (13.3)	.05
Cerebrovascular disease	11 (2.9)	1 (1.1)	10 (3.4)	.24

*Note*: age, body mass index, blood pressure, and baPWV are expressed as mean (standard deviation). Other values are expressed as numbers (%).

baPWV = brachial–ankle pulse wave velocity.

**Table 2:** Characteristics of valued life activities and brachial–ankle pulse wave velocity.

	All	baPWV < 14 m/s	baPWV ≥ 14 m/s	*P*-value
	n = 380	n = 87	n = 293	
Number of valued life activities				
1 or 2	52 (13.7)	5 (5.7)	47 (16.0)	.01
3	328 (86.3)	82 (94.3)	246 (84.0)	
Performance score	7.02 (2.1)	6.96 (2.21)	7.04 (2.07)	.75
Satisfaction score	7.21 (2.1)	7.22 (2.01)	7.21 (2.14)	.94
Frequency of valued life activities				
Monthly or yearly	136 (35.8)	35 (40.3)	101 (34.5)	.33
Daily or weekly	244 (64.2)	52 (59.7)	192 (65.5)	
Continuity of valued life activities		0	0	
<1 yr	38 (10.0)	10 (11.5)	28 (9.6)	.60
>1 yr	342 (90.0)	77 (88.5)	265 (90.4)	
Domain of valued life activities				
Self-care only	4 (1.1)	1 (1.1)	3 (1.0)	.65
Productivity only	36 (9.5)	4 (4.6)	32 (10.9)	.08
Leisure only	50 (13.4)	9 (10.3)	41 (14.3)	.38
Self-care, productivity, and leisure	48 (12.6)	11 (12.6)	37 (12.6)	.99
Self-care and productivity	47 (12.3)	12 (13.8)	35 (11.9)	.65
Self-care and leisure	59 (15.5)	12 (13.8)	47 (16.0)	.61
Productivity and leisure	135 (35.5)	38 (43.7)	97 (33.1)	.07

baPWV = Brachial–ankle pulse wave velocity.

A baPWV ≥ 14 m/s was significantly associated with the number of VLAs. In Model 1, having 3 VLAs yielded an adjusted odds ratio of 0.33 (95% confidence interval [Cl]: 0.13–0.85) (Table 3). The adjusted odds ratios for Models 2 and 3 were 0.33 (95% confidence interval [CI]: 0.12–0.86) and 0.39 (95% CI: 0.14–1.10), respectively. Regarding the domains of VLAs, the crude odds ratio was 2.54 (95% Cl: 0.87–7.41) for participants whose VLAs were only for productivity. Those whose VLAs were for leisure and productivity had a crude odds ratio of 0.64 (95% Cl: 0.39–1.04) and 0.49 (95% Cl: 0.26–0.91) for women only (Table 4).

**Table 3:** Relationship between characteristics of valued life activities and brachial–ankle pulse wave velocity ≥ 14 m/s.

	Crude odds ratio (95% Cl)	*P*-value	Model 1		Model 2		Model 3	
			Odds ratio (95% Cl)	*P*-value	Odds ratio (95% Cl)	*P*-value	Odds ratio (95% Cl)	*P*-value
Important number of valued life activities								
1 or 2	1	.02	1	.02	1	.02	1	.08
3	0.32 (0.12–0.83)		0.33 (0.13–0.85)		0.33 (0.12–0.86)		0.39 (0.14–1.10)	
Performance score	1.02 (0.91–1.14)	.75	1.03 (0.86–1.25)	.73	1.03 (0.86–1.25)	.73	1.05 (0.85–1.30)	.67
Satisfaction score	0.99 (0.89–1.12)	.94	0.97 (0.80–1.16)	.71	0.87 (0.80–1.16)	.72	0.99 (0.80–1.23)	.92
Frequency of valued life activities								
Monthly or yearly	1	.32	1	.52	1	.51	1	.28
Daily or weekly	1.28 (0.78–2.09)		1.19 (0.71–1.98)		1.19 (0.71–1.98)		1.36 (0.78–2.37)	
Continuity of valued life activities								
Less than 1 year	1	.60	1	.66	1	.66	1	.51
More than 1 year	1.23 (0.57–2.64)		1.21 (0.53–2.77)		1.20 (0.52–2.77)		1.36 (0.55–3.34)	

Model 1 = independent variables were adjusted for each other.

Model 2 = in addition to the variables in Model 1, adjustments were made for sex.

Model 3 = in addition to the variables in Model 2, adjustments were made for body mass index, smoking, alcohol intake, and underlying medical conditions.

Cl = confidence interval.

**Table 4:** Relationship between the domains of valued life activities and brachial–ankle pulse wave velocity ≥ 14 m/s.

	All		Male		Female	
	Crude odds ratio (95% Cl)	*P*-value	Crude odds ratio (95% Cl)	*P*-value	Crude odds ratio (95% Cl)	*P*-value
Self-care only	0.89 (0.09–8.66)	.92	ー (ー-ー)	ー	0.93 (0.10–9.17)	.95
Productivity only	2.54 (0.87–7.41)	.08	2.06 (0.44–9.52)	.36	3.02 (0.68–13.47)	.15
Leisure only	1.41 (0.86–3.03)	.38	1.33 (0.47–3.82)	.59	1.45 (0.47–4.49)	.52
Self-care, productivity, and leisure	0.99 (0.49–2.05)	.99	0.80 (0.29–2.20)	.67	1.21 (0.43–3.41)	.72
Self-care and productivity	0.85 (0.42–1.72)	.65	0.52 (0.15–1.85)	.31	1.06 (0.45–2.50)	.89
Self-care and leisure	1.19 (0.80–2.37)	.62	0.84 (0.33–2.17)	.72	1.66 (0.60–4.56)	.33
Productivity and leisure	0.64 (0.39–1.04)	.07	10.30 (0.45–2.37)	.94	0.49 (0.26–0.91)	.02

Self-care only is not shown because of bias in binary values in males.

Cl = confidence interval.

In a sensitivity analysis with additional adjustment for age, the association between the number of VLAs and baPWV ≥ 14 m/s was attenuated and no longer statistically significant (adjusted OR: 0.50, 95% CI: 0.17–1.45). The VIF for age was 1.05, indicating no problematic multicollinearity. Furthermore, when alternative thresholds of 16 to 18 m/s were applied, the results remained generally consistent. The odds ratios for having 3 VLAs were 0.51 (95% CI: 0.28–0.95) at 16 m/s, 0.69 (95% CI: 0.35–1.35) at 17 m/s, and 0.54 (95% CI: 0.29–0.98) at 18 m/s.

## 4. Discussion

We explored the association between VLA characteristics and baPWV in community-dwelling individuals. This study is the 1st to address arterial stiffness from the perspective of VLAs as a part of lifestyle, supported by the association between baPWV and the number and domains of VLAs. The baPWV criteria varied across studies.^[20]^ A baPWV of 14 m/s was relatively low; however, it is an effective screening tool for atherosclerosis in community dwellers,^[20]^ with approximately 23% of the participants having a baPWV < 14 m/s. In a previous survey of community-dwelling adults, the percentage of those with baPWV < 14 m/s was in the 50% range, which was higher than that in this study.^[23]^ This study included a population with a relatively high rate of arterial stiffness.

The number of VLAs was independently associated with PWV risk, even after adjusting for smoking and alcohol intake, which affect lifestyle-related and underlying diseases. Aging is a strong factor that increases baPWV.^[24]^ In this study, no adjustments were made for age to avoid over-adjustment. In the sensitivity analysis, when age was included in the adjustment, the association between the number of VLAs and baPWV was attenuated and lost statistical significance. The VIF for age was 1.05, indicating no problematic multicollinearity. This suggests that the lack of significance was not due to model instability but rather to the predominant influence of age on arterial stiffness in this older cohort. This finding underscores the significant effect of age on arterial stiffness in this cohort of older adults. Although the independent contribution of VLAs could not be confirmed after accounting for age, VLAs may still reflect lifestyle factors that interact with the aging process. Thus, VLAs may serve as complementary indicators in populations where the impact of age is less pronounced, such as middle-aged adults.

To evaluate arterial stiffness in daily life, we recommend assessing the number of VLAs. Physical activity reduces arterial stiffness^[18]^ and helps in preventing arteriosclerosis.^[25]^ In particular, aerobic exercise decreases PWV.^[26]^ Low to moderately intense physical activity is associated with arterial stiffness, and less intense physical activity may worsen it.^[8]^ Conversely, high-intensity muscle training has been reported to have negative effects,^[27]^ while resistance training in healthy participants does not alter PWV.^[28]^ Therefore, discussing atherosclerosis based solely on physical activity is insufficient. However, increased sedentary time is an independent risk factor for death and CVD.^[29]^

In this study, because VLAs are individual and diverse, and specific activities were not considered, the proportion and intensity of physical activity could not be analyzed. Therefore, it cannot be concluded that all participants engaged in physical activities. However, a greater number of VLAs may provide more opportunities to avoid sedentary behaviors. If an individual recognizes activity as important, many VLAs are intertwined and may be related to arteriosclerosis, exercise, and physical activity.

In this study, VLAs were analyzed by domain rather than specific names. The VLAs for productivity tended to be positively associated with baPWV. Conversely, the VLAs for leisure and productivity tended to be negatively associated with baPWV. A balanced approach to VLAs is important for health and can inform health promotion interventions.

The job strain index and baPWV are negatively correlated in productive jobs,^[30]^ and high job strain increases the risk of CVD.^[31]^ Although productive activity is not always exclusively a job, it is negatively associated with arterial stiffness from stress and heavy physical labor. In older adults, the frequency of performing leisure and productive activities influences physical health and depression.^[32]^ Leisure time may be a protective factor for CVD,^[7]^ but productive work may be both a risk and protective factor.^[33]^ Regarding arterial stiffness, the key is whether activities are important to the individual, and this needs to include both leisure and productive activities. Moreover, the impact of these domains on each other must be considered. The allocation between productivity and leisure may also be important for arterial stiffness.

A female-specific association was observed between VLAs and baPWV domains. The incidence of heart disease is significantly higher in postmenopausal women, strongly suggesting that menopause may be a risk factor for atherosclerosis.^[34]^ Encouraging women to engage in VLAs is important. Engaging in productivity and leisure activities was associated with lower baPWV among women. Notably, the risk of cerebrovascular disease is lower when women avoid the following 5 risks: smoking, low physical activity, inappropriate body weight, excessive alcohol consumption, and an unhealthy diet.^[35]^ Women have higher rates of insufficient physical activity than men^[36]^; however, engaging in productive activities increases total physical activity.^[37]^ In women, productivity activities such as employment, housework, and leisure positively impact subjective health perception.^[38]^ Additionally, physical activity during work and leisure reduces the risk of CVD.^[7]^ Thus, when considering arterial stiffness in women, it is necessary to account for the benefits of both productive and leisure activities.

A higher baPWV is significantly associated with a higher risk of CVD, even after adjustment for risk factors.^[39]^ Lifestyle modifications such as no smoking history, increased physical activity, maintaining an appropriate weight, alcohol restriction, and a healthy diet are effective against atherosclerosis.^[40]^ VLAs are essential for life and are integrated into daily life. Incorporating VLAS characteristics into arteriosclerosis screening may facilitate the perception of arteriosclerosis as a personal concern. Assessing the characteristics of VLAs may be useful for designing health support related to arterial stiffness.

## 5. Limitations

This study has several limitations. First, its cross-sectional design precludes causal inference; it remains unclear whether VLAs reduce arterial stiffness or if healthier individuals are more likely to engage in VLAs. Cohort studies across multiple regions are needed. Second, the reference values for baPWV vary across studies. We adopted relatively low cutoffs, which may partly explain the high prevalence of elevated baPWV. Third, VLAs were self-reported and limited to a maximum of 3, raising concerns about recall bias and restricting analyses of time use and activity intensity. Fourth, in sensitivity analyses with additional adjustment for age, the associations between VLAs and baPWV were attenuated and no longer significant. The VIF (1.05) indicated no multicollinearity, suggesting that the strong effect of age, rather than statistical instability, accounted for this attenuation. Longitudinal studies are required to clarify whether VLAs independently contribute to arterial stiffness beyond aging. Finally, while the sample size was adequate for the primary analyses, it limited the statistical power to examine subgroup effects such as gender-specific associations. Larger studies incorporating qualitative assessments of VLAs may provide deeper insights into their psychosocial dimensions and public health relevance.

## 6. Conclusion

The number of VLAs was associated with baPWV in the main analyses, and the combination of productive and leisure activities was significant for women. Although these associations weakened after adjusting for age, this likely reflects the strong influence of aging on arterial stiffness. This study is the 1st step toward understanding arterial stiffness not only from the perspective of exercise and physical activity but also from the standpoint of valued life activities. Our findings suggest that health support for individuals with arterial stiffness should not only focus on conventional risk management but also encourage engagement in activities that patients consider meaningful and important. Promoting such valued life activities may enhance adherence to lifestyle modifications and ultimately contribute to better vascular health outcomes.

## Acknowledgments

We thank the staff of the Katsuragi Town Health and Welfare Center who were involved in the data collection.

## Author contributions

**Conceptualization:** Katsushi Yokoi, Nobuyuki Miyai.

**Formal analysis:** Katsushi Yokoi, Nobuyuki Miyai.

**Funding acquisition:** Katsushi Yokoi.

**Investigation:** Nobuyuki Miyai, Masato Sakaguchi, Toshifumi Oka, Miyoko Utsumi, Megumi Nakamura.

**Project administration:** Miyoko Utsumi, Mikio Arita.

**Supervision:** Mikio Arita.

**Writing – original draft:** Katsushi Yokoi.

**Writing – review & editing:** Katsushi Yokoi, Nobuyuki Miyai, Masato Sakaguchi, Toshifumi Oka, Miyoko Utsumi, Megumi Nakamura, Mikio Arita.
